# Postoperative outcome of caesarean sections and other major emergency obstetric surgery by clinical officers and medical officers in Malawi

**DOI:** 10.1186/1478-4491-5-17

**Published:** 2007-06-14

**Authors:** Garvey Chilopora, Caetano Pereira, Francis Kamwendo, Agnes Chimbiri, Eddie Malunga, Staffan Bergström

**Affiliations:** 1 Department of Obstetrics and Gynaecology, University of Malawi, College of Medicine, Blantyre, Malawi; 2 Instituto Superior de Ciências de Saùde, Maputo, Mozambique; 3 Division of International Health (IHCAR), Department of Public Health Sciences, Karolinska Institutet, Stockholm, Sweden; 4 Centre for Reproductive Health, University of Malawi, College of Medicine, Blantyre, Malawi

## Abstract

**Background:**

Clinical officers perform much of major emergency surgery in Malawi, in the absence of medical officers. The aim of this study was to validate the advantages and disadvantages of delegation of major obstetric surgery to non-doctors.

**Methods:**

During a three month period, data from 2131 consecutive obstetric surgeries in 38 district hospitals in Malawi were collected prospectively. The interventions included caesarean sections alone and those that were combined with other interventions such as subtotal and total hysterectomy repair of uterine rupture and tubal ligation. All these surgeries were conducted either by clinical officers or by medical officers.

**Results:**

During the study period, clinical officers performed 90% of all straight caesarean sections, 70% of those combined with subtotal hysterectomy, 60% of those combined with total hysterectomy and 89% of those combined with repair of uterine rupture. A comparable profile of patients was operated on by clinical officers and medical officers, respectively. Postoperative outcomes were almost identical in the two groups in terms of maternal general condition – both immediately and 24 hours postoperatively – and regarding occurrence of pyrexia, wound infection, wound dehiscence, need for re-operation, neonatal outcome or maternal death.

**Conclusion:**

Clinical officers perform the bulk of emergency obstetric operations at district hospitals in Malawi. The postoperative outcomes of their procedures are comparable to those of medical officers. Clinical officers constitute a crucial component of the health care team in Malawi for saving maternal and neonatal lives given the scarcity of physicians.

## Background

Malawi, like many other countries in sub-Saharan Africa is facing a critical shortage of human resources across all cadres in the health sector. Due to the high cost of training medical doctors and other health personnel, the country has been faced with a chronic underproduction of health care personnel. At 1:62 000, the present doctor-to-population ratio is one of the world's lowest [1]. The Ministry of Health declared this shortage a crisis in early 2004 [2]. With the help of donor funds, the government embarked on a six year Emergency Human Resource Programme aimed at improving staff recruitment and retention in the public sector [2,3].

HIV/AIDS has taken a significant toll on health care providers. An initial Human Resources Development Plan 1999 to 2004 assumed an annual HIV/AIDS-related attrition of 2.8% [4]. However, this is thought to be an underestimate. In addition to AIDS-related deaths, health personnel have left the profession for other less risky professions for fear of being exposed to the disease. A lot of staff time has also been lost through prolonged periods of illness, funeral attendance and caring for sick relatives [3,5]. The migration of health professionals, notably doctors and nurses, to high income countries has also had a large contribution to the worsening human resource situation in countries that can least afford the depletion of human resources for health, including Malawi [5]. However this raises a conflict between the individual's right to travel and the country's need for an adequate health workforce [6].

Against this background, Malawi has to live up to the challenge of meeting the Millennium Development Goal (MDG) number 5, i.e. to reduce maternal mortality by 75% – based on the level in 1990 – within the next eight years. Success stories from Sri Lanka [7] and Malaysia [8] point to human resources as a crucial factor in reducing maternal mortality. In order to cope with the ever-increasing demand for health care, Malawi introduced a cadre of mid-level health care providers called clinical officers (COs) as early as 1976. These are non-doctors trained locally for three years. After completing a year of internship either at the central or district hospital, they (like medical officers (MOs)) are licensed to practice independently and perform major emergency and elective surgery.

Unlike in Mozambique [9,10] and Tanzania [11], the delegation of major surgery to non-doctors in Malawi has not been scientifically validated. The purpose of this study was therefore to elucidate the extent of major surgical work carried out by COs and MOs, respectively, in Malawi and to find out the quality of surgical care as observed in the postoperative outcome of patients operated upon by these two categories of staff.

## Methods

The study was conducted prospectively in all government district hospitals and CHAM (Christian Health Association of Malawi) hospitals in Malawi. A total of 38 health facilities were under study over a period of three months (October to December 2005). Four referral hospitals (Zomba Central Hospital, Mzuzu Central Hospital, Lilongwe Central Hospital and Queen Elizabeth Hospital) were not studied. They performed together an estimated 800 caesarean sections during the study period. The respective proportions carried out by COs and MOs is not known.

All women undergoing caesarean section during the study period were included in the study. The vast majority of such operations were carried out to cater for emergencies, elective caesareans constituting a small minority. We recruited one qualified nurse midwife working in the maternity unit as a research assistant at each of the hospitals. All women undergoing caesarean section were followed up from the time the decision to do a caesarean section was made until discharge from hospital. Women were asked to come back for review seven days after discharge. A structured data collection sheet was used to retrieve information on admission diagnosis, indication for surgery, preoperative condition, designation of surgeon and type of surgery.

We also assessed the competence of the two types of professionals that were the performing surgeons, by noting information about the institution at which they did their internship as well as the number of years of practice each of them had after a completed internship. Although medical doctors play a role in the training of COs, much of the on-the-job practical experience is passed on from CO to CO, since the newly qualified COs often are sent straight to the district hospital for their internship to fill the gaps in human resources. The senior COs therefore take the responsibility of teaching, as in most cases there is no doctor available at the station.

Outcome measures included neonatal condition, immediate and 24 hour maternal condition, post-operative fever, wound sepsis and mortality. Outcomes of surgery by COs were compared with those of surgery performed by MOs.

Data was entered in SPSS statistical package and the unpaired chi square test was used to test for significance of the differences in outcome between COs and MOs. When appropriate, Fisher's exact test was used.

## Results

A total of 2131 emergency obstetric operations were performed in the 38 centres during the study period (Table 1). Of these, 1875 (88%) were done by COs while 256 (12%) were done by MOs. COs performed as many as 93% of these surgical operations in government district hospitals and 78% in CHAM hospitals.

**Table 1:** Type of operation and category of surgeon (C/S = caesarean section)

**Type of operation**	**Clinical officers**	**Medical officers**	**Total**
C/S only	1569 (89.5%)	185 (10.5%)	1754 (100.0%)
C/S + subtotal hysterectomy	11 (57.9%)	8 (42.1%)	19 (100.0%)
C/S + total hysterectomy	7 (70.0%)	3 (30.0%)	10 (100.0%)
C/S + repair of uterine rupture	59 (89.4%)	7 (10.6%)	66 (100.0%)
C/S + bilateral tubal ligation	224 (80.9%)	53 (19.1%)	277 (100.0%)
Not indicated	5 (100.0%)	0 (0.0%)	5 (100.0%)
Total	1875 (88.0%)	256 (12.0%)	2131 (100.0%)

The distribution of interventions was comparable in the two groups of surgeons. Of all 1875 operations carried out by COs 1569 (84%) were CSs only, while this figure was somewhat less for MOs (72%) (Table 1). Hysterectomies occurred in around 1% of all interventions by COs, while this figure was 4% among MOs. More tubal ligations occurred among MO interventions (20%) than among CO interventions (12%). The diagnoses prescribing surgery were cephalopelvic disproportion, obstructed labour, previous caesarean section, fetal distress, suspected ruptured uterus, ante partum haemorrhage, cord prolapse, prolonged labour, breech presentation and eclampsia (Table 2). The distribution of these diagnoses in the two categories of surgeons did not differ significantly.

**Table 2:** Indications motivating surgery

**Indication**	**Number of cases**
Cephalopelvic disproportion or obstructed labour	1230
Previous caesarean section	452
Fetal distress	264
Suspected ruptured uterus	87
Antepartum haemorrhage	77
Cord prolapse	62
Failure to progress	60
Breech in primigravida	53
Eclampsia	49

Of the operations (n = 256) performed by MOs, 199 (77.7%) were done by MOs who had done their internship at the central hospital. Of these 256 interventions, 55 (21.5%) were by foreign doctors who had had their internship outside the country. Of the operations (n = 1,875) performed by COs, only one fourth were done by COs with internship at the central hospital. Half of all the CO operations were performed by COs with internship at district hospital level (Table 3).

**Table 3:** Institution where the clinical officers did their internship against the

**Institution of intership**	**Number of operations**	**Proportion of operations done by clinical officers(%)**
District Hospital	948	50.5
CHAM Hospital	476	25.4
Central Hospital	447	23.8
Outside Malawi	1	0.1
Not indicated	4	0.2
Total	1876	100.0

The post-internship surgical experience had a duration of four years or more in 44% of COs and in 59% of MOs, while the figures for three years or less were 46% and 37%, respectively (Table 4). It should, however, be noted that as much as 9% of COs admitted no post-internship surgical experience at the moment of interview.

**Table 4:** Duration of surgeons' post-internship surgical practice

**Duration**	**Clinical officers**	**Medical officers**	**Total**
Four years or more	832 (44.4%)	151 (59.0%)	963 (46.1%)
Two to three years	456 (24.3%)	61 (19.9%)	507 (23.8%)
Less than one year	401 (21.4%)	44 (17.2%)	445 (20.9%)
None	175 (9.3%)	-	175 (8.2%)
No information	11 (0.6%)	10 (3.9%)	21 (1.0%)
Total	1875 (100.0%)	256 (100.0%)	2131 (100.0%)

The outcome figures for newborns were similar in the two groups (Table 5). The same overall pattern was also noted for maternal outcomes, being almost identical by comparison (Table 6). Of the patients, 83% stayed in hospital for two days or less prior to surgery. There was no significant difference in the number of days required for hospitalization in the two groups of surgeons. Unknown HIV status was almost universal (98%) and 65% received preoperative antibiotics. The immediate postoperative outcome was evaluated, followed by a repeat evaluation at 24 hours after surgery. A gross categorization was established (Tables 6 and 7), indicating no major difference between cases operated upon by COs and MOs, respectively. The subjectivity of these evaluations is a limitation of this study; however, the more specific classification elaborated in Table 8 would seem to confirm the findings in Tables 6 and 7.

**Table 5:** Postoperative neonatal outcomes in relation to category of surgeon

**Neonatal outcome**	**Clinical officers**	**Medical officers**	**Total**
Alive and well	1604 (85.5%)	213 (83.2%)	1817 (85.2%)
Alive and unwell	70 (3.7%)	9 (3.5%)	79 (3.7%)
Stillbirth	160 (8.5%)	29 (11.3%)	189 (8.9%)
Early neonatal death	41 (2.2%)	4 (1.6%)	45 (2.1%)
No information	-		1 (0.0%)
Total	1875 (100.0%)	256 (100.0%)	2131 (100.0%)

Difference not statistically significant, p = 0.709

**Table 6:** Immediate post-operative maternal general condition in relation to category of surgeon.

**Condition**	**Clinical officer**	**Medical officers**	**Total**
Fair	1700 (90.7%)	235 (91.8%)	1935 (90.8%)
Sick	105 (5.6%)	17 (6.6%)	122 (5.7%)
Very sick	27 (1.4%)	3 (1.2%)	30 (1.4%)
No information	43 (2.3%)	1 (0.4%)	44 (2.1%)
Total	1875 (100.0%)	256 (100.0%)	2131 (100.0%)

Difference not statistically significant, p = 0.786

**Table 7:** Maternal general condition 24 hours after operation in relation to category of surgeon

**Condition**	**Clinical officers**	**Medical officers**	**Total**
Fair	1765 (94.1%)	243 (94.9%)	2008 (94.2%)
Sick	59 (3.1%)	9 (3.5%)	68 (3.2%)
Very sick	20 (1.1%)	1 (0.4%)	21 (1.0%)
No information	31 (1.7%)	3 (1.2%)	34 (1.6%)
Total	1875 (100.0%)	256 (100.0%)	2131 (100.0%)

Difference not statistically significant, p = 0.564

**Table 8:** Specific maternal post-operative outcomes in relation to category of surgeon

**Condition**	**Clinical officers**	**Medical officers**	**p value**
Fever	388 (20.7%)	56 (21.9%)	0.364
Wound infection	137 (7.3%)	14 (5.5%)	0.994
Wound dehiscence	40 (2.1%)	4 (1.6%)	0.315
Need for re-operation	28 (1.5%)	5 (2.0%)	0.364
Maternal death	22 (1.2%)	1 (0.4%)	0.292

There were numerically more maternal deaths in the CO group (n = 22/1875; 1.2%) than in the MO group (n = 1/256; 0.4%) but the difference is not statistically significant by Fisher's exact test. Broken down by type of intervention, the distribution of maternal deaths was: 4/18 (22%) died after CS and hysterectomy, whereas only 11/1569 (0.7%) died after CS only. Of uterine rupture cases, 6/59 (10%) died postoperatively (Table 9). The case fatality rates by specific preoperative morbidity in this group of CS patients are presented in Table 10, indicating that eclampsia and clinical signs of uterine rupture had the highest rates at around 6 %

**Table 9:** Maternal deaths by operative procedure

**Procedure**	**Number of deaths(n = 23)**	**Number undergoing procedure**	**Procedure-related case fatality rate (%)**
C/Section only	11	1569	0.7
C/S + Subtotal hysterectomy	2	11	18.2
C/S + Total hysterectomy	2	7	28.6
C/S + Repair of uterine rupture	6	59	10.2
C/S + Tubal ligation	1	224	0.4
No information	1	-	-

**Table 10:** Maternal death by pre-operative diagnosis.

**Diagnosis**	**Number of deaths(n = 23)**	**Number with diagnosis**	**Case fatality rate**
Eclampsia	3	52	5.7%
Obstructed labour	9	580	1.6%
Previous C/Section(s)	2	460	0.4%
Suspected ruptured uterus	5	87	5.7%
Fetal distress	1	264	0.4%
CPD	3	650	0.5%

## Discussion

The problem of high maternal mortality ratios and perinatal mortality rates is endemic in most low-income countries. Multiple factors are involved in this sustained scenario. Such factors include unavailability of a sound health care system with adequate essential supplies; facilities for emergency obstetric care, both basic and comprehensive; social, cultural and political factors; as well as the absence of skilled attendants at the time of delivery [11,12]. In the face of the current human resource crisis, each country, poor or rich, needs to have a national workforce plan shaped to its situation and crafted to address its health needs [5].

For many years Malawi has been dependent on COs for the provision of health services both in the rural and urban areas of the country due to the chronic shortage of medical doctors. This may be considered a variant of a two tier system of training where some health personnel are trained to a basic level and therefore are more likely to be retained in the country [13,14]. Our study found that as many as 93% of major emergency obstetric operations in government district hospitals were done by COs and this includes surgery on complicated conditions. This is similar to earlier findings by Fenton et al., where 65% of caesarean sections at central and district hospitals were done by COs [15,16]. It is noteworthy that a similar study in Mozambique revealed the figure of 92% [Pereira et al, unpublished results].

The profile of patients operated on by COs was found to be comparable to that of patients operated on by MOs, with similar indications for surgery in the two groups of surgeons. During the study it was found that 50% of the surgeries were done by COs who had done their internship at the district hospital. In some instances, COs undergoing internship were doing caesarean sections on their own. It might be argued that, even if COs have well documented manual skills in performing even major surgery, they may not have skills in diagnostic accuracy comparable to those of MOs. This aspect is not investigated. The issue of preoperative diagnostic skills will therefore be the focus of our forthcoming research.

Monitoring and evaluating quality of care is subject to a certain degree of subjectivism. It may be argued that the positioning of a local nurse midwife with well known competence as an 'impartial' (though non-blinded as far as type of surgeon was concerned) individual might imply a bias. Although assessment of postoperative outcome is largely a subjective matter, we attempted to make it as objective as possible by asking them to collect such objective data as blood pressure level, pulse rate, amount of vaginal bleeding, post operative pyrexia, wound infection, wound dehiscence and need for re-operation in addition to the general clinical condition of the patient.

The case fatality rates (CFRs) of a few defined morbidities, suspected ruptured uterus, eclampsia and obstructed labour, are well above the level WHO has suggested, less than 1% [17]. It should be noted, however, that the WHO target refers to the "crude" CFR, implying all deaths divided by all morbidities, which we consider gives too blunt a picture of the quality of emergency care. We consider morbidity-specific CFR a more appropriate measure of quality of care than the "crude" CFR.

The major cause of maternal death (where clearly identifiable) was sepsis. This is similar to the findings of the confidential inquiry into institutional maternal deaths in the southern region of Malawi by Ratsma [18].

Other factors than events surrounding the surgery come into play. Most of these patients will have spent a number of days on the way to hospital, some even coming from abroad. In addition, unknown HIV status was almost universal and only slightly more than half of the patients received preoperative antibiotics.

## Conclusion

Clinical officers constitute a key category of health workers to save women's lives by providing advanced emergency obstetric care. They perform the bulk of emergency obstetric operations at district hospitals in Malawi. The postoperative outcomes of their procedures are comparable to those of medical officers. However, in order to sustain and further enhance quality of surgical care by COs, it would be of value that all COs – like all MOs – should do their internship in surgery at central hospitals to ensure a uniform base of competence and capacity. Given the scarcity of physicians in Malawi, COs have a vital role to play for decades to come in the provision of life-saving major surgery, particularly at district level.

## Competing interests

The author(s) declare that they have no competing interests.

## Authors' contributions

GCC planned the study with CP. CP provided the background methodology and contributed with the design in collaboration with SB. FK, AC and EM contributed in preparing the documents and the protocol for implementing the study. CP, GCC, SB and EM prepared and completed the final analysis of data.
